# The utility of wearable electroencephalography combined with behavioral measures to establish a practical multi-domain model for facilitating the diagnosis of young children with attention-deficit/hyperactivity disorder

**DOI:** 10.1186/s11689-024-09578-1

**Published:** 2024-11-11

**Authors:** I-Chun Chen, Che-Lun Chang, Meng-Han Chang, Li-Wei Ko

**Affiliations:** 1 Department of Physical Medicine and Rehabilitation, Ton-Yen General Hospital, Hsinchu, Taiwan; 2 Department of Early Childhood Education and Care, College of Human Ecology, Minghsin University of Science and Technology, Hsinchu, Taiwan; 3 Artise Biomedical Co., Ltd, Hsinchu, Taiwan; 4 Department of Psychiatry, Ton-Yen General Hospital, Hsinchu, Taiwan; 5 Department of Electronics and Electrical Engineering, Institute of Electrical and Control Engineering, National Yang Ming Chiao Tung University, Hsinchu, Taiwan; 6 Center for Intelligent Drug Systems and Smart Bio-devices (IDS2B), College of Biological Science and Technology, National Yang Ming Chiao Tung University, Hsinchu, Taiwan; 7 Department of Biomedical Science and Environment Biology, Drug Development and Value Creation Research Center, Kaohsiung Medical University, Kaohsiung, Taiwan

**Keywords:** Attention-deficit/hyperactivity disorders (ADHD), Preschool children, Electroencephalography (EEG), Conners’ kiddie continuous performance test second edition (K-CPT-2), Rating scales, Machine learning, Deep learning, Wearable technology, Artificial intelligence (AI)

## Abstract

**Background:**

A multi-method, multi-informant approach is crucial for evaluating attention-deficit/hyperactivity disorders (ADHD) in preschool children due to the diagnostic complexities and challenges at this developmental stage. However, most artificial intelligence (AI) studies on the automated detection of ADHD have relied on using a single datatype. This study aims to develop a reliable multimodal AI-detection system to facilitate the diagnosis of ADHD in young children.

**Methods:**

78 young children were recruited, including 43 diagnosed with ADHD (mean age: 68.07 ± 6.19 months) and 35 with typical development (mean age: 67.40 ± 5.44 months). Machine learning and deep learning methods were adopted to develop three individual predictive models using electroencephalography (EEG) data recorded with a wearable wireless device, scores from the computerized attention assessment via Conners’ Kiddie Continuous Performance Test Second Edition (K-CPT-2), and ratings from ADHD-related symptom scales. Finally, these models were combined to form a single ensemble model.

**Results:**

The ensemble model achieved an accuracy of 0.974. While individual modality provided the optimal classification with an accuracy rate of 0.909, 0.922, and 0.950 using the ADHD-related symptom rating scale, the K-CPT-2 score, and the EEG measure, respectively. Moreover, the findings suggest that teacher ratings, K-CPT-2 reaction time, and occipital high-frequency EEG band power values are significant features in identifying young children with ADHD.

**Conclusions:**

This study addresses three common issues in ADHD-related AI research: the utility of wearable technologies, integrating databases from diverse ADHD diagnostic instruments, and appropriately interpreting the models. This established multimodal system is potentially reliable and practical for distinguishing ADHD from TD, thus further facilitating the clinical diagnosis of ADHD in preschool young children.

**Supplementary Information:**

The online version contains supplementary material available at 10.1186/s11689-024-09578-1.

## Introduction

Artificial intelligence (AI) and machine learning (ML)-based approaches for analyzing medical information and biological signals have progressively developed in recent decades. In clinical research regarding neurodevelopmental disorders, neuroscientists have utilized AI and ML techniques to survey the brain-mind-behavioral relationship, focusing on neurobiological features of the brain [1]. Among multimodal data — such as genetic, neuroimaging, and clinical behavioral data —electroencephalography (EEG) recording is non-invasive, easy to implement, cost-effective, more tolerant of motion artifacts, and provides excellent temporal resolution [2–5]. These advantages make EEG particularly suitable for studying infants and young children, especially when investigating neural activity during cognitive processing, which is the most challenging. Moreover, a recent systematic review [6] indicated that techniques for identifying and classifying mental health issues in children and adolescents extensively rely on physiological signals, largely due to their safety. Notably, EEG signals have been most frequently employed for identifying various mental developmental problems.

Attention-Deficit/Hyperactivity Disorder (ADHD) is an early-onset neurobehavioral disorder that can influence children’s learning, social performance, and well-being from preschool age [7–9]. The American Academy of Pediatrics, “Clinical Practice Guideline: Diagnosis and Evaluation of the Child with ADHD” addresses the evaluation, diagnosis, and treatment of ADHD in children aged 4 to 18 years [9]. The diagnostic criteria for ADHD in preschool children require that core symptoms present across more than one setting, which may be challenging to access when the child does not undergo care or education outside their home [10]. Normally, this information is obtained through clinical interviews with parents, direct observation of the child in a clinical setting, and symptom checklists from the aforementioned situations. Neuropsychological measures can support the clinical diagnosis of ADHD [11]. As with school-age children, a multi-method, multi-informant approach that evaluates behavioral functioning across multiple settings is emphasized for evaluating and diagnosing ADHD in preschoolers [10, 12].

Given the complexity and challenges of ADHD diagnosis, numerous studies have considered alternative approaches to improve the efficiency of early diagnosis through AI techniques, namely ML and deep learning (DL) methodologies to establish and promote the accuracy of ADHD diagnosis. In reviewing AI studies in ADHD [13], the authors noted a lack of focus on the utility of wearable technologies for ADHD diagnosis. They proposed that AI research should incorporate multiple types of datasets derived from diagnostic instruments to develop a robust clinical judgment assistive scheme regarding ADHD, both inside and outside clinical settings. In alignment with this viewpoint, a recent systemic review summarized the results obtained for automated ADHD detection in children and adolescents. It indicated EEG as the most widely used physiological biomarker, with a yearly gradual increase in its application for ADHD detection in children. However, several limitations in this field were identified, including the reliance on a single dataset for model development, a lack of explainability, and the absence of signal fusion [6].

Most AI studies on the automated detection of ADHD have used a single type of data such as MRI, EEG, questionnaires, or game-based tests. To the best of our knowledge, few studies focus on preschool children and employ multiple modalities to develop their models. However, recent studies have begun to combine more than one evaluation tool to establish AI-assisted diagnosis systems for ADHD [14–18]. Since the ‘gold standard’ for diagnosing ADHD comprises a combination of neuropsychological tests, behavioral rating scales from different observers, clinical interviews, and examination of intervention outcomes, a multi-method, multi-informant approach that evaluates manifestation functioning across more than one setting is optimal for evaluating ADHD in preschoolers [12, 19]. Single-modality AI approaches for ADHD diagnosis have been surveyed widely in the past. Nevertheless, a multimodal method may be more appropriate due to the heterogeneous clinical characteristics of ADHD.

Consequently, this study aims to respond to the aforementioned issues by using a wearable wireless EEG device combined with standard diagnostic tools, including the continuous performance test (CPT) and the ADHD-related symptom scoring inventory to create a reliable AI-detection system. We anticipate that this multimodal system could serve as a practical, robust, and clinically interpretable tool for assisting in the diagnosis of ADHD at the preschool age.

## Materials and methods

### Participants

This study enrolled 78 young children, all of whom were in kindergarten and had not yet started primary school at the time of their participation. Of these, 43 were diagnosed with ADHD (35 boys, mean age: 68.07 ± 6.19 months) and 35 had typical development (TD) (25 boys, mean age: 67.40 ± 5.44 months). This sample excluded children with significant neurological disorders, congenital syndromes, chromosomal and genetic disorders, hearing or visual impairments, autism spectrum disorders, intellectual disabilities, or any other psychiatric disorders. All participants with ADHD were diagnosed according to the Diagnostic and Statistical Manual of Mental Disorders, 5th Edition (DSM-V) [7] criteria by the certified child and adolescent psychiatrist in a clinical setting.

Additionally, all participating children and parents were informed of details regarding the experiment by the project leader, and written informed consent was obtained from the parents before their recruitment. The study protocol was approved by the Research Ethics Committee of the National Health Research Institutes in Taiwan (EC1070401-F).

### Clinical neuropsychological measurement

#### Intelligence test and ADHD-related behavioral rating scales

The Taiwanese version of the Wechsler Preschool and Primary Scale of Intelligence, Fourth Edition was used by qualified psychologists to evaluate all participating children’s cognitive functioning in a clinical setting [20]. To assess ADHD symptoms in all subjects, both the parent and teacher versions of the Disruptive Behavior Disorder Rating Scale (DBDRS) were completed by the subjects’ parents and kindergarten teachers. The DBDRS evaluates two major ADHD-related dimensions — (a) inattention and (b) hyperactivity-impulsivity — and is appropriate for use with young children [21]. The DBDRS is reliable and valid in preschool children across a variety of study samples [22, 23].

#### Conners’ kiddie continuous performance test, second edition

The Conners’ Kiddie Continuous Performance Test Second Edition (K-CPT-2) [24], a commercially available computerized instrument, was used to assess the attention-related performance of the participating children. The K-CPT-2 incorporated five blocks (sets of trials), with one block consisting of two sub-blocks where each sub-block included 20 trials. After completing a total of 200 trials, which took 7.5 min, nine main standardized scores were generated to allow the clinician to interpret the children’s attentional problems. As defined by a mean of 50 and an SD (standard deviation) of 10, higher scores indicate poorer performance, except hit reaction time (HRT).

In these main indices, *d’* (detectability) measures the ability to discriminate between targets and non-targets. Omissions represent the rate of missed targets, while commissions denote the rate of incorrect responses to non-targets. Perseverations refer to anticipatory, repetitive, or random responses detected in less than 100ms after the stimulus. HRT calculates the mean response speed for all non-perseverative responses throughout the test. The HRT SD and variability reflect the consistency of the response speed. HRT Block Change assesses sustained attention by calculating the slopes of change in reaction time across the five blocks of the test, and HRT Inter-stimulus Interval (ISI) Change measures the slope of reaction time between longer and shorter ISIs, indicating the vigilance performance of subjects [24].

### Experimental paradigm (Fig. 1)

In the experimental procedure, the participating children were instructed to sit comfortably in front of a computer screen at the clinical environment, namely within the outpatient clinic. The assessor encouraged the children to relax, ensured their comfort, and then recorded one minute of baseline eye-open (EO) resting EEG data. Next, the assessor provided test instructions and guided the children through practice trials. Finally, task EEG data was recorded during the formal K-CPT-2 session for 7.5 min after practice [25–28].

**Fig. 1:**
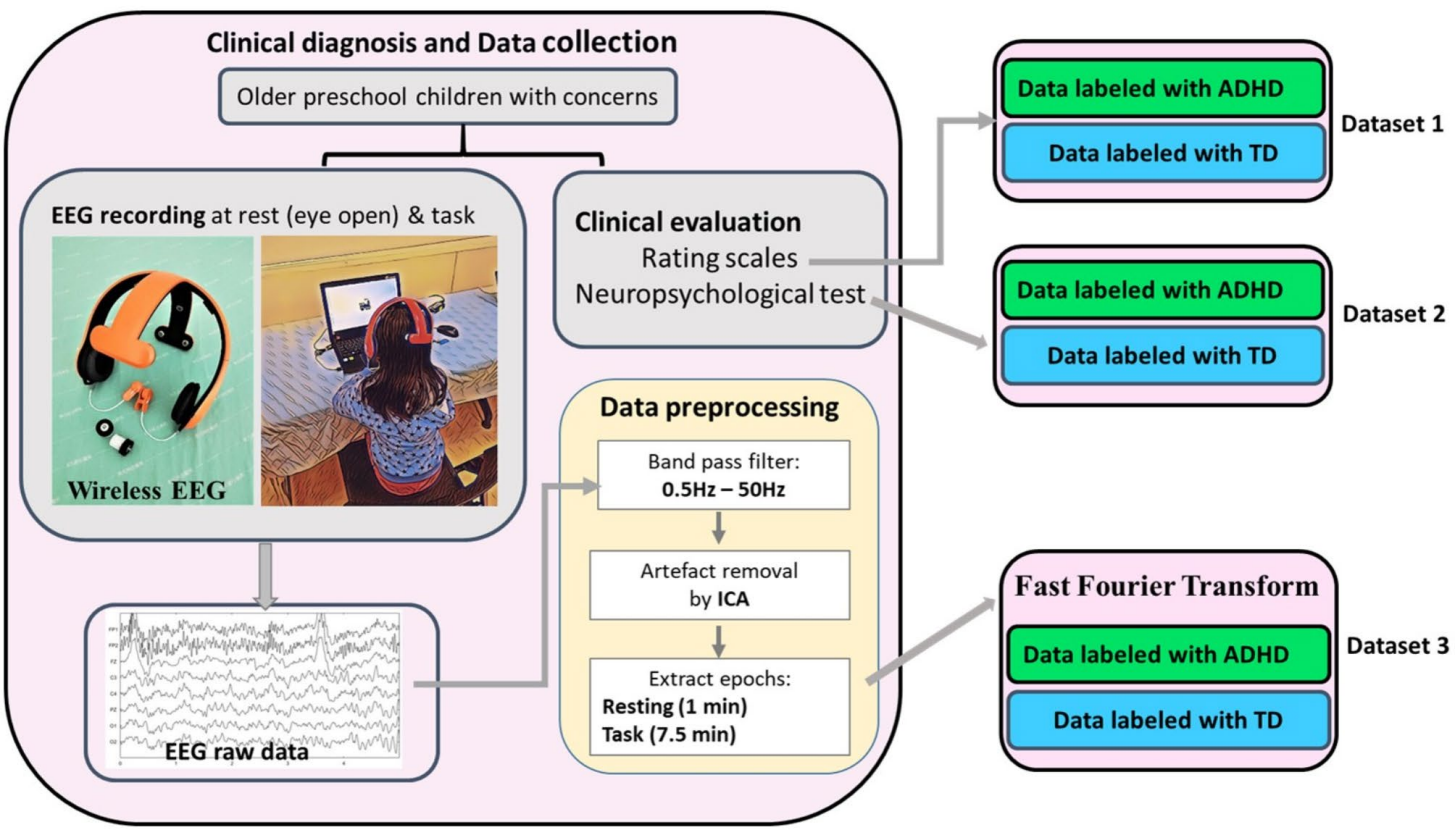
Graphical display of wireless EEG data collection and dataset generation

### EEG recording via wearable technology (Fig. 1)

In this study, an 8-channel wearable wireless EEG system [27, 29] (Mindo BR8, the Brain Research Center of National Chiao Tung University, Hsinchu, Taiwan) was used to record EEG data (one-minute EO resting and 7.5 min task) from all participating children. The raw EEG signals were collected from eight electrode sites on the scalp (Fp1, Fp2, Fz, C3, C4, Pz, O1, and O2), with impedance set below 100 κOhms. The EEG data was recorded at a rate of 1000 samples per second and referenced to linked earlobes [27, 29]. This device, noted for its comfort and ease, has proven highly applicable for clinical use in young children [25–27].

### EEG signals processing (Fig. 1)

#### Artifact removal

To enhance signal quality, EEG experts employed two methodologies, which were meticulously delineated in our preceding research [25–27]. Initially, the raw signal was filtered using basic finite impulse response filters with a passband of 0.5–50 Hz. Subsequently, all trials were subjected to a visual inspection, during which components containing electrical, muscular, or eye movement artifacts were identified and manually removed using Independent Component Analysis (ICA). ICA is a widely utilized blind source separation technique that divides multivariate signals into regions of interest and those containing artifacts [30]. The threshold for the number of components to be removed was set at 2/8 (25%), a more conservative approach that aimed to retain as many signals as possible. Following the application of the ICA technique, components associated with artefacts were identified and removed. The remaining components, which were free from artefacts, were then used to compose clean EEG data for further analysis. In conclusion, the mean percentage of valid ICA components retained was greater than 75%. The entire resting data segment was retained for 60 s following preprocessing, and data preprocessing was conducted using EEGLAB in the MATLAB toolbox.

#### Datasets for input

According to the data recording conditions, one minute of resting power and 7.5 min of task-related K-CPT-2 power were separately processed for further computation.

#### Fast fourier transform (FFT)

To convert the raw time domain signal into frequency band domains ranging from 0 to 50 Hz (Hz), the Welch function of FFT was adopted in this study. Five different frequency bands were defined: delta (1–4 Hz), theta (4–8 Hz), alpha (8–13 Hz), beta (13–30 Hz), and low gamma (30–50 Hz). We investigated the total artifact-free power spectral density of the EEG data for each participant using the short-time Fourier transform spectrogram function of the signal processing toolbox in MATLAB [27].

#### Normalization of the input data

Min-max normalization was applied to the absolute power of data.

### EEG-based classification model to identify ADHD

#### Deep learning model

To identify the most optimal model for analyzing the data, bidirectional LSTM (Long Short-Term Memory) was implemented. This recurrent neural network architecture builds upon the traditional LSTM model by processing input sequences in both forward and backward directions. In a standard LSTM, information flows from the past to the future, sequentially processing the input sequence. In contrast, a bidirectional LSTM captures dependencies from both past and future contexts [31, 32].

The bidirectional LSTM comprises two LSTM layers: one processes the input sequence from left to right (forward LSTM), while the other processes the sequence in the reverse direction (backward LSTM) [33, 34]. Each LSTM layer contains multiple memory cells or units that maintain a hidden state, representing information from the previous elements in the sequence. These hidden states are updated based on the current input and the previous hidden state using a set of learned weights [35].

After processing the input sequence in both directions, the output of the bidirectional LSTM is typically obtained by concatenating the forward and backward hidden states at each time step. This combined representation integrates information from both past and future contexts and can be further used for classification, sequence labeling, or sequence generation [33, 34].

Bidirectional LSTMs are particularly effective for tasks where the current prediction depends on both past and future contexts, such as in natural language processing applications like machine translation, sentiment analysis, speech recognition, and named entity recognition [31, 36–38]. By leveraging both forward and backward information, bidirectional LSTMs improve the model’s ability to capture complex patterns and dependencies in sequential data.

In this present study, the EEG datasets from each participating child are analyzed individually since the proposed LSTM network is trained to identify ADHD on a patient-specific basis. EEG features for signal analysis are extracted from every 0.001-second segment of EEG data (i.e. 60,000 resting and 450,000 task EEG data samples) to be used as input to the LSTM classifier. (Supplementary Fig. 1)

#### Hyperparameter tuning

Using grid search for hyperparameter tuning, we selected the most optimal pattern as our training model with a hyperparameter setting. (Supplementary Fig. 2)

#### Validation method

To evaluate the quality of the predictive EEG-based model, we allocated 70% of the data to training and 30% to testing. In validation, we used 90% of the data for training and 10% for validation.

### The classifiers for neuropsychological measurement

Regarding clinical neuropsychological measures (DBDRS and K-CPT-2 scores), decision tree (DT) and random forest (RF) classifiers were used for this study. Specifically, two clinical measures were utilized to construct model 1 (DBDRS) with a DT approach and model 2 (K-CPT-2) with RF classifiers.

### Performance evaluation and feature analysis of models

To evaluate the performance of the prediction models, we used precision, recall, and F1 scores to display the results corresponding to the three individual models and the ensemble model.

For clinical neuropsychological and EEG data, we aimed to clarify which features are most important for differentiation. Using permutation methods, we determined the order of important features from the inner loop in the individual models.

### Ensemble of classifiers (Fig. 2)

To obtain a unified classification system, we established an ensemble of classifiers by combining the earlier-generated diverse predictive models. Specifically, we trained three different classifiers on independent datasets, further deriving three independent predictive models: “Predictive model #1” trained on DBRS features, “Predictive model #2” trained on K-CPT-2 features, and “Predictive model #3” trained on EEG features.

**Fig. 2:**
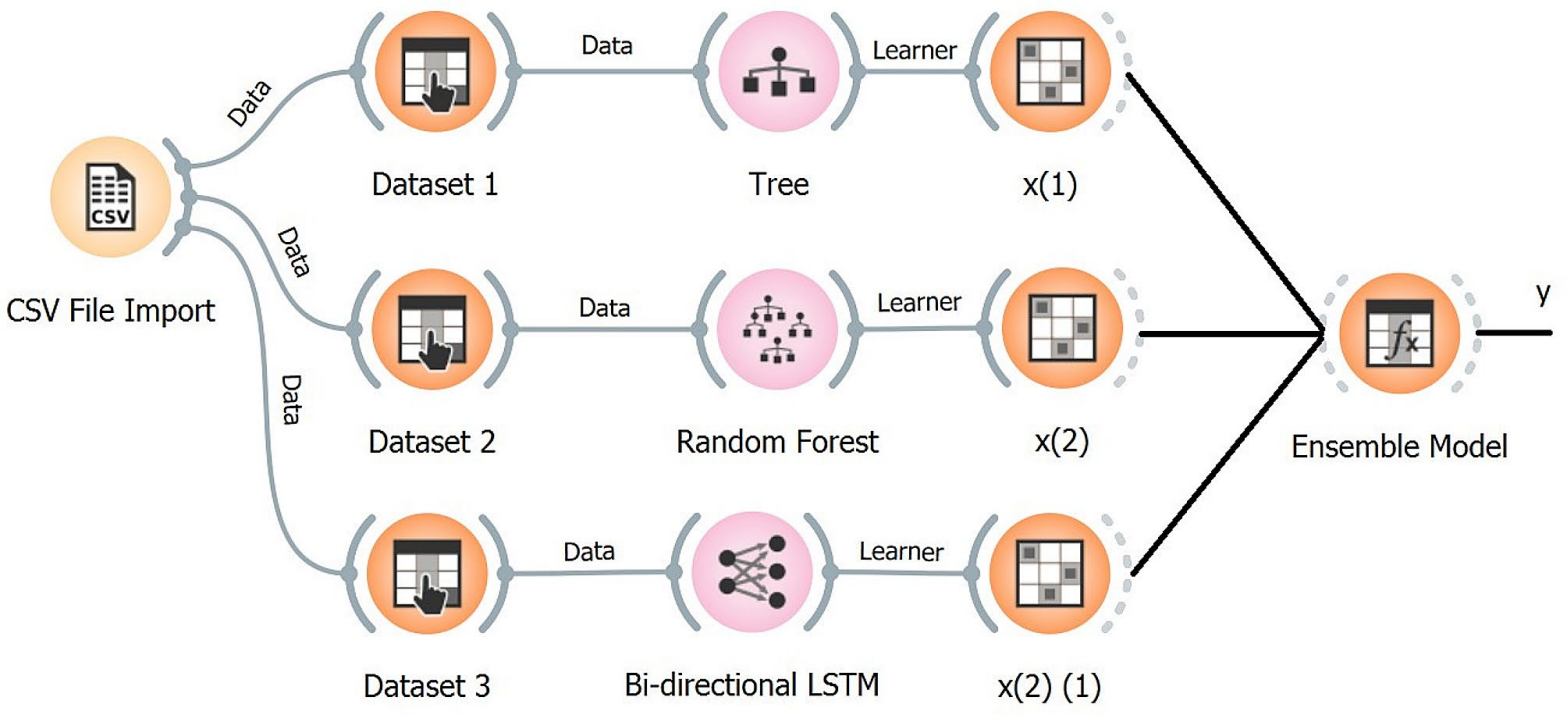
The ensemble model proposed in this research. The ensemble model consists of 3 basic classifiers: decision tree, random forest, and bidirectional LSTM models

Figure 2 illustrates the ensemble model proposed in this research. This ensemble model consists of 3 basic classifiers: DT, RF, and bidirectional LSTM model.

$$\:{f}_{\left(x\right)\:}=\frac{1}{3}{x}_{1}+\frac{1}{3}{x}_{2}+\frac{1}{3}{x}_{3}$$

To predict the target label class, we use an aggregator function where, $\:{f}_{\left(x\right)}$is an aggregate function of the ensemble models,$\:\:{x}_{1},\:{x}_{2},\:{x}_{3}$ are the predicted outputs for each model, and the threshold value is 0.5. Additionally, the same weight is employed for each model. If the value of $\:{f}_{\left(x\right)}$ is greater than 0.5 then the predicted value is 1, and vice versa.

$$\:\widehat{y}=\left\{\begin{array}{c}1,\:if\:{f}_{\left(x\right)}\ge\:0.5\\\:0,\:\:\:otherwise\end{array}\right.$$

### Statistical analysis

Demographic and clinical measures were compared between the two groups using independent t-tests, and a comparative analysis of categorical variables (e.g., sex) was performed using a chi-squared test.

## Results

### Demographic and clinical measurement data

Table 1 compares the demographic and clinical measurements between the two groups. There were no significant differences regarding the mean age, sex distribution, full-scale intelligence quotient, and the verbal comprehensive index.

**Table 1:** Comparison of demographic characteristics and clinical measures for total sample, ADHD, and TD

Mean (SD)	ADHD	TD	*P*-value
	*n* = 43	*n* = 35	
Age-months	68.07 (6.19)	67.40 (5.44)	0.617
Gender (male/female)	35/8	25/10	0.418
FSIQ	94.49 (14.03)	96.45 (3.96)	0.546
VCI	98.05 (12.83)	96.79 (3.18)	0.676
DBDRS-P-i	13.86 (4.41)	9.57 (4.92)	< 0.001**
DBDRS-P-h	13.14 (5.90)	8.11(5.21)	< 0.001**
DBDRS-T-i	15.58 (5.18)	8.79 (6.07)	< 0.001**
DBDRS-T-h	13.35 (5.85)	6.71 (7.20)	< 0.001**
K-CPT-2 scores			
d’	51.74 (7.46)	48.77 (6.50)	0.068
Omission	51.09 (8.00)	48.09 (7.61)	0.096
Commission	50.16 (8.85)	47.66 (8.83)	0.217
Perseveration	49.07 (6.23)	47.83 (6.99)	0.41
HRT	58.28 (8.24)	55.17 (6.62)	0.075
HRT SD	52.05 (8.45)	47.94 (6.49)	0.021*
Variability	51.72 (9.51)	48.00 (6.49)	0.052
HRT block change	50.53 (8.51)	48.74 (5.63)	0.288
HRT ISI change	52.44 (8.89)	47.69 (8.62)	0.020*

** *P* < 0.01, * *P* < 0.05; ADHD: attention-deficit/hyperactivity disorder, TD: typical development, SD: standard deviation, FSIQ: full-scale intelligence quotient, VCI: verbal comprehension index, DBDRS-P-i: Disruptive Behavior Disorder Rating Scale parent version inattentiveness dimension, DBDRS-P-h: Disruptive Behavior Disorder Rating Scale parent version hyperactivity dimension, DBDRS-T-i: Disruptive Behavior Disorder Rating Scale teacher version inattentiveness dimension, DBDRS-T-h: Disruptive Behavior Disorder Rating Scale teacher version hyperactivity dimension, d’ detectability, K-CPT-2: Conners’ Kiddie Continuous Performance Test Second Edition, HRT: hit reaction time, ISI: inter-stimulus interval

In terms of the clinical measurements, significant differences were observed in specific DBDRS and the K-CPT-2 scores between the two groups. The group of children with ADHD received higher scores than those with TD in DBDRS-inattentive dimension by both parents (13.86 ± 4.41 versus 9.57 ± 4.92, *p* < .001) and teachers (15.58 ± 5.18 versus 8.79 ± 6.07, *p* < .001). Similarly, the DBDRS-hyperactive dimension by both parents (13.14 ± 5.90 versus 8.11 ± 5.21, *p* < .001) and teachers (13.35 ± 5.85 versus 6.71 ± 7.20, *p* < .001) were also rated higher. In respect of the K-CPT-2, the group with ADHD underperformed (scoring higher) than TD in HRT SD (52.05 ± 8.45 versus 47.94 ± 6.49, *p* = .021) and HRT ISI change (52.44 ± 8.89 versus 47.69 ± 8.62, *p* = .020) (Table 1).

### Performance evaluation of models

Supplementary Fig. 3 shows the training results of model 3, which was generated using EEG data. Our model achieved a maximum training accuracy of 0.9472 (94.72%) and a maximum validation accuracy of 87.68%. Epochs are represented 10 times for each scale, meaning that our model attained maximum and stable accuracy after 120 epochs. Furthermore, during the testing phase, we used 5-fold cross-validation to ensure the robustness of the results and found that our model acquired an accuracy of 0.95 (95%) (Table 2).

**Table 2:** Performance evaluators of the ADHD and TD classification models

Model #	Precision	Recall	F1-Score	Accuracy
1. Decision Tree	0.912	0.909	0.909	0.909
2. Random Forest	0.923	0.922	0.922	0.922
3. Bi-Direct. LSTM	0.950	0.940	0.950	0.950
**Ensemble Model**				**0.974**

Concerning models 1 and 2, the precision, recall, and F1 score are listed in Table 2.

### Ensemble model performance

In particular, the accuracy of the final ensemble model is 0.974 (Table 2).

### Significant features analysis

**Fig. 3:**
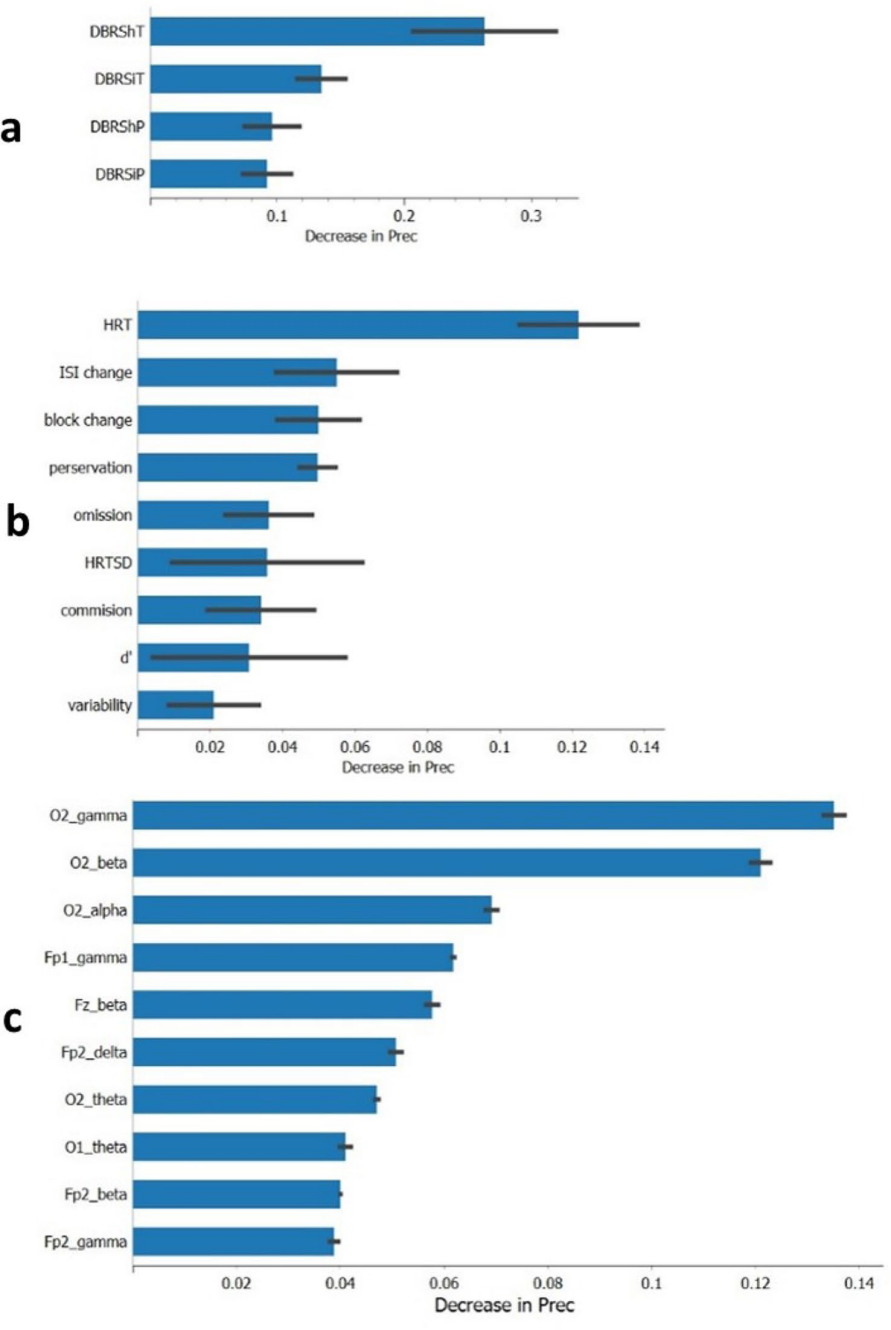
The ranks of important features in predictive model #1 - trained by decision tree (**a**), in predictive model #2 - trained by random forest (**b**), in predictive model #3 - trained by bidirectional LSTM

Figure 3(a) highlights that the most important feature from the dataset of model #1 is DBRShT, which shows the effect of a decrease in precision of about 0.25 ± 0.05. DBRSiT is the next most important feature, leading to a precision decrease of approximately 0.13 ± 0.01. Specifically, the absence of DBRSiT in model #1 would result in a reduction of precision by 0.25 ± 0.05, while the absence of DBRSiT from model #1 would lead to a decrease in precision by 0.13 ± 0.01.

Figure 3(b) emphasizes that the most important feature of model #2 is HRT, which decreases the precision by about 0.12 ± 0.02. HRT ISI change follows with a precision decrease of 0.06 ± 0.02, and block change ranks third, reducing the precision by 0.05 ± 0.02. The rest of the feature values are shown in Fig. 3(b). Specifically, the absence of HRT in model #2 would result in a precision reduction of 0.12 ± 0.02.

Figure 3(c) shows that the most important features from the data set of model #3 are O2 low-gamma, O2 beta, and O2 alpha, which decrease the precision by around 0.14, 0.12, and 0.07, respectively. In other words, the absence of O2 low-gamma, O2 beta, and O2 alpha data from model #3 would result in a decrease in precision by 0.14, 0.12, and 0.07, respectively.

## Discussion

This study combines behavioral scales, computerized attention tests, and wireless EEG data to develop a multimodal system. This system offers clinicians a reliable, diverse, and clinically understandable scheme for assisting in the diagnosis of ADHD in young children. In this ensemble model, the DBDRS provides subjective observations, within a period, from main caregivers, and the K-CPT-2 scores offer objective behavioral quantification. Furthermore, the EEG recordings indicate brain signals related to attention. These three dimensions of datasets represent distinct aspects of assessment, aligning with the clinical guidelines of multi-methods regarding preschool ADHD diagnosis. Our results answer the key three objectives in ADHD-related AI studies: the usability of wearable technologies, combining databases from different ADHD diagnostic instruments, and adequate interpretability of our ML and DL models.

In ADHD-related research, brain MRI data are mainly used to develop neuroimaging-based AI models [1, 13]. In terms of physiological signals, EEG is the most widely studied instrument for surveying automated ADHD diagnostic systems, demonstrating favorable performance with accuracy results exceeding 80–90% through ML, DL, or novel algorithms/approaches [39–51]. Other biological data have attempted to identify genetic biomarkers of ADHD using ML and DL [13]. Externalizing symptom and behavior measures using survey forms [52], performance tests [53], and motion data [54] have been studied for ADHD classification among children. In general, these studies focused on a single form of dataset. However, it is well-established that the diagnosis of preschool ADHD should rely on multiple assessment approaches.

Few studies have made efforts to adopt multiple datasets from varied modalities [14–18]. Crippa et al. [14] investigated the capability of multi-domain profiles including blood fatty acid testing, neuropsychological assessments, and near-infrared spectroscopy measures, to accurately identify school-aged children with ADHD. In addition, Yoo et al. [16] and Kautzky et al. [15] analyzed neuroimaging and genetic data to propose multivariate classification models for ADHD and healthy controls. Due to the invasive nature of blood testing and the less naturalistic experimental setting of MRI, these methods may raise concerns about clinical practicality and compliance in preschool children. Two additional studies employed two neuroimaging datasets to combine features derived from functional near-infrared spectroscopy and EEG, resulting in enhanced classification accuracy [18]. Similarly, the utilization of EEG and magnetoencephalography connectivity features led to improved classification accuracy [17]. However, these features are primarily from a single category, namely, neural-based signals. The present study addresses these aforementioned limitations by using the wireless EEG-integrated game-like test, which is specifically non-invasive, clinical acceptable, and offers a more diverse perspective.

Our results identify significant features from 3 different models consisting of scores of DBDRS rated by teachers and HRT in K-CPT-2, as well as EEG O2 low-gamma band power. These findings are clinically interpretable and compatible with previous literature. Tandon and Pergjika [55] indicated an increased judgment confidence in the improper behaviors of preschoolers if teachers reported the presence of symptoms. Rather than relying on parents’ feedback on their kindergarten-level children, their teachers can make more accurate comparisons with their peers who do not exhibit ADHD-related behaviors, thereby providing more valuable information. The results of our model #1 align with previous literature supporting that teacher ratings may be more informative than parent ratings [56, 57]. Regarding reaction time, children with ADHD are typically less accurate, slower, and exhibit more variability compared to their age-matched peers in the review articles [58, 59]. The results of our model #2 are consistent with this finding, indicating that HRT is the most significant feature.

The EEG feature analysis in model #3 reveals that the resting band power of low-gamma and beta are the most important indicators. While most ADHD-related EEG literature has examined the power of theta and the theta-beta ratio [2, 3, 60–62], few studies surveyed the gamma power for the small amplitude, and the importance of high-frequency EEG oscillations in cognitive function and neurodevelopmental disorders, which is often underestimated compared to low-frequency oscillations [63, 64]. Herrmann and Demiralp [63] stated that the hyperactive behavior of ADHD patients results from a neuronal hyper-excitation reflected by enhanced gamma activity. In terms of beta power, Clarke et al. [65, 66] found that a subgroup of children with ADHD had significantly excessive beta oscillations and were further determined to be the combined type of ADHD in boys than girls. In the same cases with gamma response, this EEG profile of excess beta activity was perceived as hyper-arousal. Our feature analysis results can attest to previous conclusions. Additionally, to interpret the context of resting condition and occipital brain region, we speculated that it is related to the eye open and visual stimulation status during the baseline of the experiment.

We have attempted to make the features of our predictive models clear since the process of ML and DL algorithm is highly complex, and clinicians usually perceive these models as “black boxes”, which are difficult to comprehend and utilize. Given the variations in biomarkers and features across studies, it should be realized that these approaches used for establishing the AI models are multivariate analysis methods, and can undercover complicated patterns of differences that univariate statistical methods cannot efficiently differentiate [14].

In this present study, wearable technology is employed to record the biological signal, in contrast to the lengthy procedure of preparing traditional wet electrode EEG systems. Also, the design of this device features convenience to use, which promotes the acceptance of some impatient and agitated children. Moreover, most of the child participants reacted comfortably and with little restraint during the recording of measurements. In conclusion, this technology allows for the monitoring of brain dynamics in child subjects during task performance through enhancing cooperation and naturalistic situations. We propose that this wireless semidry-electrode EEG recording system is appropriate as a data acquisition device, especially for young children. Furthermore, we have established a robust AI-facilitating clinical decision system to identify ADHD, both in and out of clinical settings.

Despite overcoming the challenges of collecting task-related brain signals in young children and analyzing the data of moderate sample size, there are several notable limitations. First, the relatively small sample size of the participating groups of children remains a main concern. Some difficulties were encountered in the recruitment of subjects. Despite the provision of comprehensive information, some parents declined to participate in the experiment, citing concerns about the safety of the EEG examination. In addition, incomplete data must be excluded due to the children’s high level of restlessness during the test. Second, the findings of this work may be specific to the age group used in our sample (i.e., 5-7-year-old children with ADHD), limiting the generalizability of the predictive models to all preschool children. Moreover, there is a possibility of selection bias towards children who were uncooperative.

## Conclusion

This present study is an investigation focusing on young children, an under-researched area. Therefore, a reliable model is established using non-invasive and wearable instruments to obtain multi-method datasets, and further reveals characteristic features for assisting ADHD diagnosis and interpreting underlying brain-behavior relationships. The findings suggest that teacher rating scores, HRT in K-CPT-2, and occipital high-frequency EEG band power are significant features in predicting abnormality and that the ensemble model can achieve an accuracy of 0.974. In summary, this established multimodal system demonstrated the potential utility of multiple perspective assessment in discriminating ADHD from TD and explaining possible neural behavioral mechanisms associated with preschool ADHD.

Further direction may integrate additional diverse non-invasive and efficient device measures, such as actigraphy and eye tracker, to enhance the evaluation dimensions and develop an instant online test system. This will prove beneficial for clinical practice and for addressing the needs of young children suspected of having ADHD.

## Electronic supplementary material

Below is the link to the electronic supplementary material.

Supplementary Material 1

Supplementary Material 2

Supplementary Material 3

## Data Availability

The datasets analyzed during the current study are available from the corresponding author on reasonable request.
