# Supplementary figures and images for: The utility of wearable electroencephalography combined with behavioral measures to establish a practical multi-domain model for facilitating the diagnosis of young children with attention-deficit/hyperactivity disorder
Source: J Neurodev Disord. 2024 Nov 11;16:62. doi: 10.1186/s11689-024-09578-1 (PMC11552361; doi:10.1186/s11689-024-09578-1)

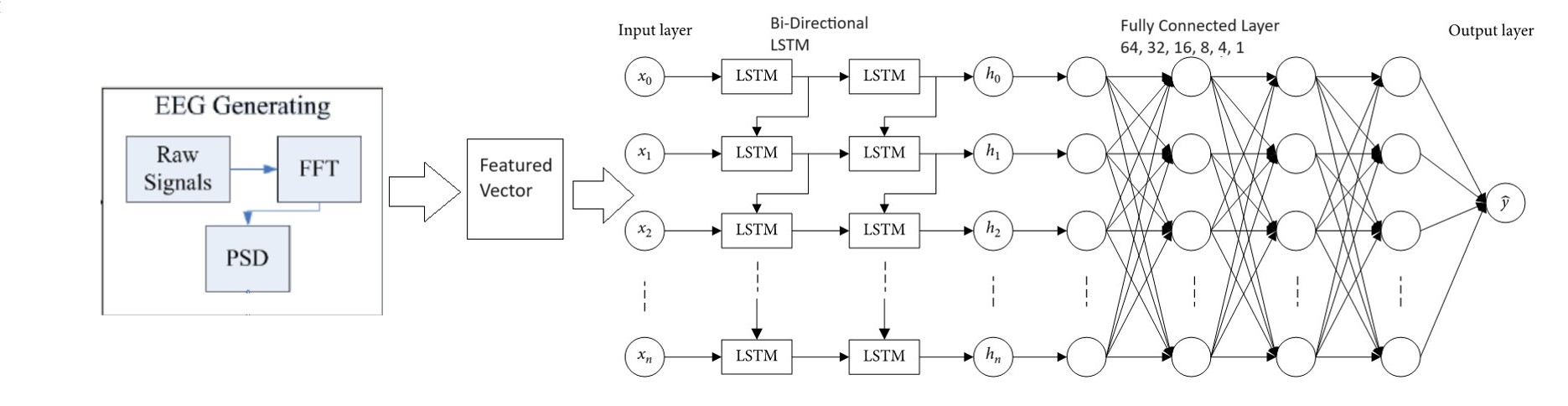

Supplement: Supplementary file 1 — Supplementary Material 1 [file 11689_2024_9578_MOESM1_ESM.jpg]

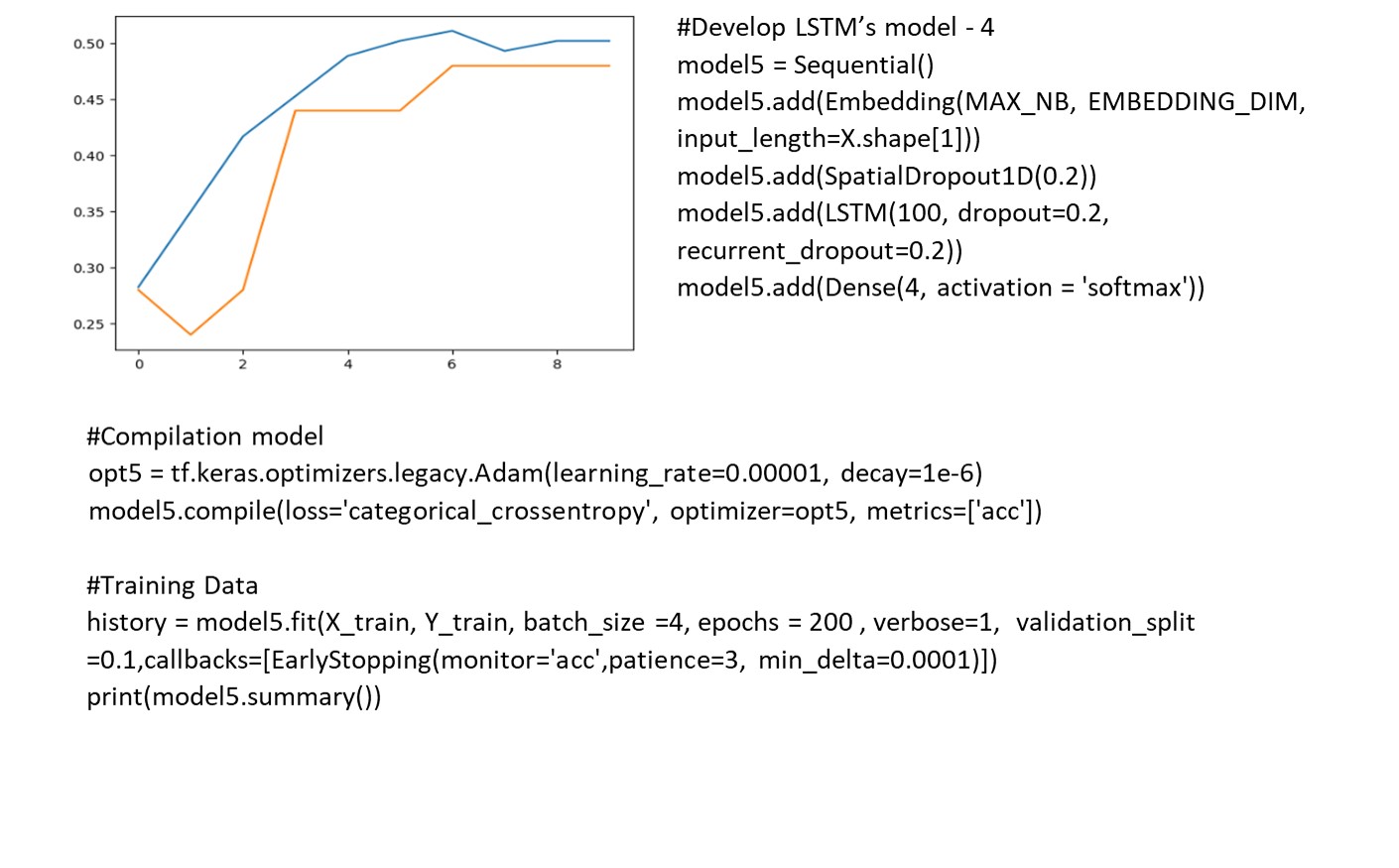

Supplement: Supplementary file 2 — Supplementary Material 2 [file 11689_2024_9578_MOESM2_ESM.jpg]

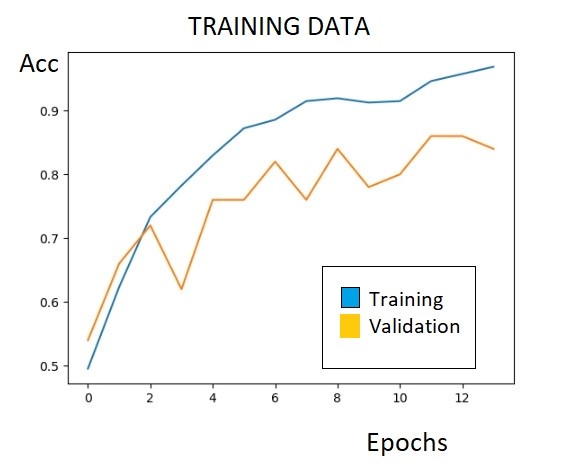

Supplement: Supplementary file 3 — Supplementary Material 3 [file 11689_2024_9578_MOESM3_ESM.jpg]
